# The Bullhorn and Beyond: Evidence‐Based Review and Clinical Recommendations for Lip Lift Techniques

**DOI:** 10.1111/jocd.70703

**Published:** 2026-02-23

**Authors:** Oskar Komisarek, Łukasz Banasiak, Vanessa Olichwer, Paweł Burduk

**Affiliations:** ^1^ Department of Otolaryngology, Phoniatrics and Audiology, Faculty of Medicine, Collegium Medicum Nicolaus Copernicus University in Toruń Bydgoszcz Poland; ^2^ The Regional Specialist Hospital in Olsztyn Olsztyn Poland; ^3^ Student Scientific Association of Maxillofacial Surgery, Faculty of Medicine Collegium Medicum, Nicolaus Copernicus University in Toruń Bydgoszcz Poland

**Keywords:** facial aesthetic surgery, lip lift, lip proportions, perioral rejuvenation, smile design, systematic review, upper shortening

## Abstract

**Background:**

The upper lip plays a central role in facial harmony and youthful appearance. Aging‐related changes include elongation of the philtrum, thinning of the vermilion, and reduced maxillary incisor show. Surgical lip lift shortens the cutaneous upper lip and enhances vermilion exposure, yet patient selection and outcome assessment remain non‐standardized.

**Objectives:**

To critically evaluate current evidence on surgical lip lift techniques, including the classical subnasal bullhorn approach and its modifications, and to provide evidence‐based recommendations for anatomy‐driven technique selection.

**Methods:**

A PRISMA 2020–compliant systematic review was conducted, with protocol registration in PROSPERO. Studies included adult patients (≥ 18 years) undergoing lip lift for aesthetic or aesthetic‐functional indications. Prospective and retrospective clinical studies and case series (≥ 5 patients) published between 2017 and 2025 were identified through database searches. Data extraction focused on surgical technique, morphometric outcomes, patient satisfaction, and complications. Meta‐analysis was not feasible due to heterogeneity. Methodological quality was assessed using the MINORS instrument and JBI checklists.

**Results:**

Seven studies comprising 1754 patients were included. Subnasal bullhorn techniques and their modifications reduced philtral length from 14.0–14.5 mm to 10.8–12.0 mm, increased vermilion height from 5.0–6.0 mm to 7.0–9.0 mm, and improved maxillary incisor show from 1.5–2.0 mm to 3.5–5.0 mm. Patient satisfaction was high (mean GAIS score 4.4/5). Complications were predominantly mild and transient, with revision rates of 0.6%–6.7%. Overall study quality was moderate.

**Conclusions:**

Surgical lip lift is a safe and effective procedure for restoring upper‐lip proportions. Technique selection should be individualized, and further prospective studies with standardized outcome measures are needed.

**Trial Registration:**

CRD420251232005

## 1 Introduction

Facial aesthetics increasingly focus on proportion, balance, and natural expression rather than isolated correction of age‐related changes. The midface and perioral region are central to the perception of youthfulness and emotional vitality, as the upper lip strongly influences facial harmony and attractiveness [1, 2, 3]. Even subtle variations in philtral length, vermilion show, and dental exposure can alter the impression of age and expression. The lip lift, defined as a surgical procedure designed to shorten the cutaneous portion of the upper lip and increase vermilion visibility, has emerged as a reliable tool for restoring lower facial balance. In contrast to *upper lip shortening* performed in orthognathic or reconstructive procedures, the aesthetic lip lift specifically targets soft‐tissue rejuvenation while preserving natural mobility and expressiveness. Global data from the International Society of Aesthetic Plastic Surgery (ISAPS) report a continuous rise in demand for facial aesthetic surgery, with procedures involving the perioral region showing a notable increase of nearly 30% between 2018 and 2023 [4]. These epidemiologic trends reflect a growing patient preference for individualized, lasting, and anatomically guided interventions aimed at achieving natural facial rejuvenation.

The upper lip undergoes characteristic structural and morphological changes with aging. Sun et al. demonstrated that loss of dermal elasticity, soft‐tissue atrophy, and resorption of maxillary support contribute to elongation of the philtrum, thinning of the vermilion, and diminished incisal show [5]. These changes disrupt the vertical proportions of the midface and lead to a fatigued or aged appearance. Quantitative evidence provided by Sleilati and Chalhoub confirmed that the philtral height increases by a mean of 5.5 mm between youth and older adulthood, from approximately 14 mm to nearly 20 mm in female patients [6]. This elongation correlates strongly with age and produces measurable effects on facial balance. Ethnic and gender variations also influence perioral morphology—female lips generally present greater vermilion definition loss, while skeletal and soft‐tissue support differ across populations [5, 6]. Such variability underscores the importance of objective preoperative analysis and individualized planning when addressing lip aging.

The development of lip lift techniques has paralleled advances in facial aesthetic surgery, progressing from superficial excisional approaches to more anatomically precise procedures. The subnasal bullhorn lift, first introduced by Reed and Millard in the early 1980s, remains the foundational concept in upper‐lip rejuvenation. The characteristic “bullhorn”‐shaped excision along the nasal base effectively conceals the scar within the alar creases and enables predictable vertical shortening. Wollina emphasized its safety, reliability, and reproducibility in both rejuvenative and corrective indications [7]. Subsequent refinements introduced by Guerrissi and De Benito optimized scar concealment, incision curvature, and soft‐tissue redraping. Gomi described several modern modifications—including the Italian lift, corner lip lift, and extended alar base approach—that improved lateral lip contour and tension distribution [1]. Yamin further synthesized the literature on surgical upper‐lip enhancement, demonstrating consistent improvement in philtral proportions and patient satisfaction across various methods [2]. Recent advances, such as the *deep‐plane lip lift* popularized by Talei, incorporate limited sub‐SMAS dissection and muscular suspension to enhance longevity and maintain natural dynamic motion [8]. Compared with temporary injectable augmentation, surgical lip lift offers a durable, structure‐based correction of vertical imbalance and restoration of incisal display.

Despite increased clinical adoption, there remains a lack of consensus on patient selection, measurement methodology, and outcome assessment. Zhao et al. reviewed contemporary lip lift techniques and highlighted major heterogeneity in surgical indications, quantitative evaluation, and follow‐up protocols [3]. Similarly, Nagy et al. demonstrated that although subnasal lifting provides lasting rejuvenation, long‐term outcome measures and complication rates are inconsistently reported [9]. This lack of standardization limits the comparability of published data and prevents the formulation of evidence‐based guidelines. Moreover, few studies integrate objective morphometric assessment with clinical decision‐making. Common complications such as hypertrophic scarring, asymmetry, and over‐shortening are discussed inconsistently, and few series stratify results by gender, ethnicity, or surgical modification. Consequently, methodological rigor and reproducibility remain key unmet needs in the field.

The present systematic review aims to synthesize and critically appraise current evidence on surgical lip lift procedures, encompassing traditional subnasal bullhorn, deep‐plane, and modified approaches. By integrating quantitative data with clinical insights, this study provides evidence‐based recommendations for selecting appropriate techniques according to anatomical subtype and patient goals. To our knowledge, this represents one of the first PRISMA 2020–compliant systematic reviews combining methodological analysis with practical clinical guidance in upper‐lip rejuvenation. The synthesis establishes a framework for standardized evaluation and supports the development of reproducible protocols in aesthetic lip surgery, bridging the gap between surgical evidence and clinical application.

## 2 Materials and Methods

### 2.1 Study Design and Registration

This systematic review was conducted in accordance with the PRISMA 2020 guidelines. The review protocol was prospectively registered in PROSPERO. The objectives, eligibility criteria, search strategy, and outcome measures specified in the registered protocol were fully adhered to, and no deviations from the original protocol occurred during the conduct of this review. The PROSPERO registration was completed prior to data extraction and synthesis.

### 2.2 Eligibility Criteria

Eligibility criteria were defined using the PICOS framework.

#### 2.2.1 Population

Adult patients (≥ 18 years) undergoing upper lip lift for aesthetic or combined aesthetic‐functional indications. Studies involving congenital deformities, trauma, oncologic reconstruction, or pediatric patients were excluded. Intervention: Surgical upper lip lift procedures, including subnasal “bullhorn,” Italian lip lift, corner lip lift, alar base extensions, and deep‐plane modifications. Comparison: Comparative and non‐comparative clinical studies were eligible. Outcomes: Morphometric or aesthetic results (philtral height, vermilion show, incisal show), patient satisfaction, complications, and revision rates. Study Design: Prospective or retrospective clinical studies, and case series with ≥ five patients. Case reports, reviews, editorials, letters, and conference abstracts were excluded.

Only articles published in English between January 2017 and October 2025 were included. This time window was chosen to capture modern lip lift techniques and contemporary outcome reporting, as significant refinements (e.g., deep‐plane approaches, standardized morphometric assessment) emerged after 2017.

### 2.3 Information Sources

A comprehensive search was conducted in PubMed/MEDLINE, Scopus, Web of Science, and the Cochrane Library. Google Scholar was used to identify additional gray literature. The final search was performed on October 31, 2025.

Backward and forward citation tracking of the included full‐text studies was performed to identify additional eligible publications. Reference lists of key articles (e.g., Gomi, Yamin, Zhao, Sleilati, Nagy) were manually screened.

### 2.4 Search Strategy

Search strategies incorporated MeSH/Emtree terms and free‐text keywords. Searches were performed in PubMed/MEDLINE, Scopus, Web of Science, the Cochrane Library, and Google Scholar to identify potentially relevant gray literature. A representative PubMed search string was:

*("lip lift" OR "subnasal lift" OR* "bullhorn lip lift" *OR* "deep‐plane lip lift" *OR* "Italian lip lift" *OR* "corner lip lift"*) AND ("aesthetic" OR "facial rejuvenation" OR "perioral")*

No study design filters were applied at the search stage. Searches were limited to articles published in English between January 2017 and October 2025, in accordance with the predefined protocol. The complete, reproducible search strategies for all databases, including exact search strings and applied limits, are provided in Supporting Information Table S1, in compliance with PRISMA 2020 reporting requirements.

### 2.5 Temporal Scope and Rationale

The literature search was restricted to studies published between January 2017 and October 2025. This temporal limitation was intentionally selected to focus on contemporary lip lift techniques and outcome reporting standards rather than on historical descriptions of the procedure.

Although seminal descriptions of the upper lip lift—including the bullhorn and central techniques—were published in earlier decades, these studies were predominantly descriptive, technique‐oriented reports that lacked standardized outcome measures, objective anthropometric assessments, and validated patient‐reported outcome instruments.

From approximately 2017 onward, the literature demonstrates a methodological shift characterized by the introduction of deep‐plane release concepts, refined suspension techniques, and systematic reporting of outcomes using objective measurements (e.g., millimeter‐based changes, nasolabial angle assessment) and validated scales such as GAIS or sGAIS. Contemporary studies also place greater emphasis on complication profiles, scar assessment, and patient satisfaction using reproducible metrics.

As the primary objective of the present systematic review was to synthesize evidence on modern lip lift techniques and their reported aesthetic outcomes, inclusion of older technique‐description studies without comparable outcome data would have limited the interpretability and clinical relevance of the synthesis. Seminal historical contributions are therefore acknowledged in the background and discussion sections but were not included in the systematic analysis.

This temporal restriction was predefined in the review protocol and applied consistently across all databases to ensure methodological coherence and comparability of reported outcomes.

### 2.6 Study Selection

Search results were exported to a spreadsheet for manual deduplication. Two reviewers independently screened titles and abstracts according to predefined eligibility criteria. Full‐text articles were then evaluated independently by both reviewers. Discrepancies were resolved through discussion and consensus; no third reviewer was required.

**FIGURE 1:**
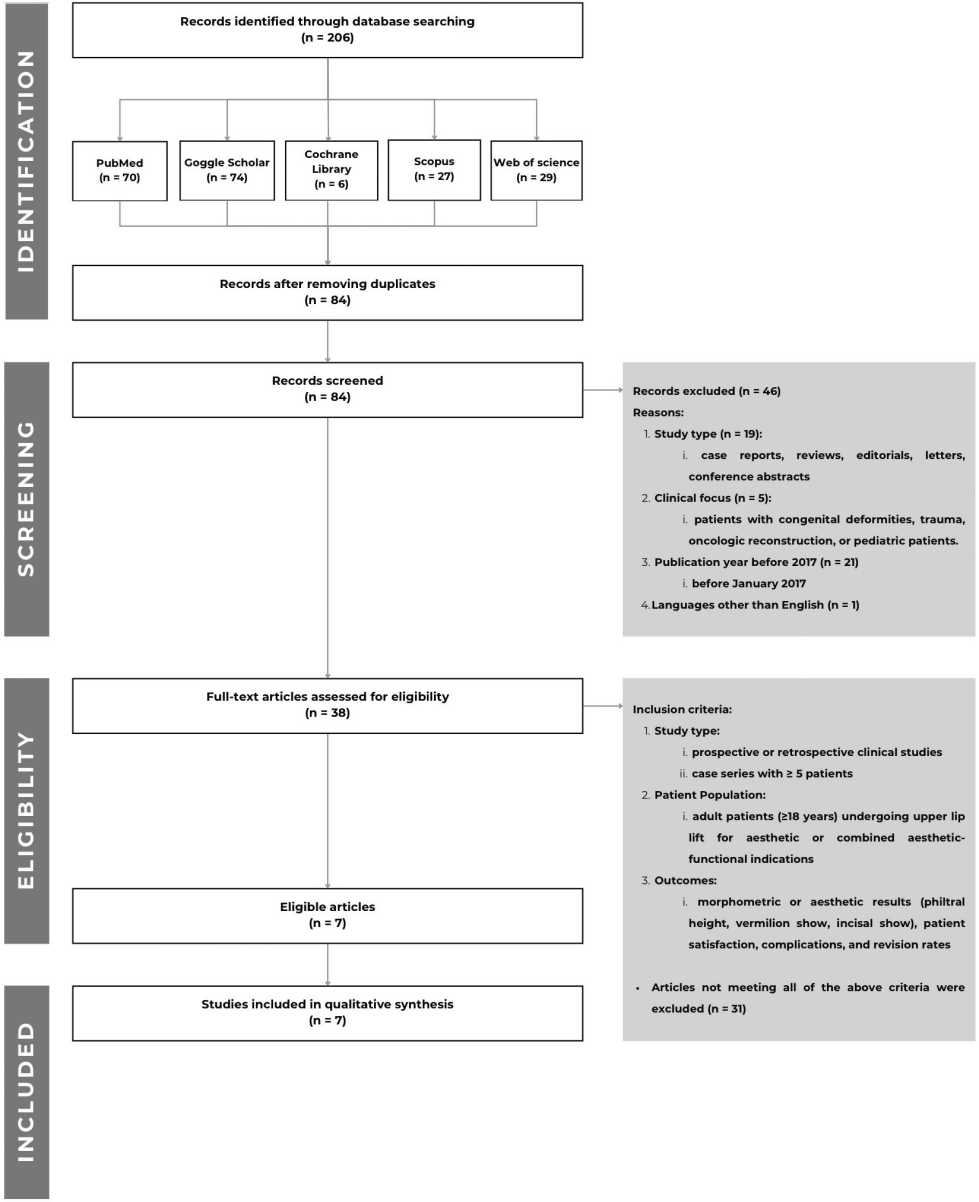
PRISMA 2020 flow diagram of study selection.

The PRISMA 2020 flow diagram illustrating the study selection process is provided in Figure 1.

### 2.7 Data Extraction

A structured data extraction sheet was developed for this review. For each included study, the following variables were collected:

- study characteristics (author, year, country, design),
- patient demographics (sample size, mean age, sex distribution),
- surgical technique (bullhorn, Italian, corner, deep‐plane, modified approach),
- operative details (incision design, tissue handling, anesthesia),
- quantitative outcomes (philtral shortening, vermilion height changes, incisal show),
- complications and revision rates,
- aesthetic assessments and follow‐up duration.

Two reviewers performed data extraction independently. Missing data were not imputed.

### 2.8 Risk of Bias and Methodological Quality

Methodological quality was assessed using:

- MINORS (Methodological Index for Non‐Randomized Studies) for observational studies, − Joanna Briggs Institute (JBI) Checklist for Case Series for descriptive series.

MINORS scores ranged from 0 to 16 for non‐comparative studies and 0–24 for comparative studies. Studies were classified as low (< 50%), moderate (50%–75%), or high quality (> 75%). JBI assessments were summarized descriptively. Reviewers conducted assessments independently and resolved differences through discussion.

A summary of quality appraisals is presented in Table 1.

**TABLE 1:** Characteristics of included studies.

Study	Design	Sample (*N*)	Mean age (range)	Follow‐up
Comparative studies				
Buhsem 2023 [10]	Comparative	28 (14 + 14)	G1: 55 years (49–66); G2: 54 years (48–65)	6 months
Mahmood 2025 [11]	Comparative	243 (Tri‐Lift 193; Control 50)	Tri‐Lift: 28 years; Control: 32.48 years	6 months
Single‐arm studies				
Talei 2019 [8]	Single‐arm	823	NR	> 3 months
Jeong 2020 [12]	Single‐arm	498 (434 F, 64 M)	F: 38.99 years; M: 34.19 years	1, 3, 6 months
Jung 2019 [13]	Single‐arm	30 (25 F, 5 M)	48.7 years (28–65)	≥ 12 months
Pascali 2021 [14]	Single‐arm	45 (40 F, 5 M)	F: 35 years (25–45); M: 38 years (26–50)	12 months
Seo 2023 [15]	Single‐arm	87 (77 F, 10 M)	35.6 years (19–67)	Mean 24.2 months (6–31.2)

### 2.9 Data Synthesis

Due to heterogeneity in study design, surgical techniques, outcome measures, and follow‐up periods, a meta‐analysis was not feasible. A qualitative synthesis was therefore performed. Studies were grouped by technique (bullhorn, Italian, corner, deep‐plane), and outcomes were summarized descriptively with emphasis on morphometric trends, complication patterns, and aesthetic results.

### 2.10 Ethical Considerations

As this review analyzed previously published data, ethical approval and informed consent were not required. The study adhered to the standards of the International Committee of Medical Journal Editors (ICMJE). The authors confirm that the ethical policies of the Journal of Cosmetic Dermatology have been adhered to.

### 2.11 Reporting Standards

This review was conducted and reported in accordance with PRISMA 2020. The PRISMA checklist is available in the Supporting Information.

## 3 Results

### 3.1 Study Selection

Seven studies met the inclusion criteria and were included in the qualitative synthesis (Figure 1). Collectively, the studies involved 1754 patients undergoing upper lip lift procedures using subnasal bullhorn lifts, deep‐plane suspension lifts, central lip lifts, indirect subnasal lifts combined with rhinoplasty, and corner mouth lifts. Two studies had a comparative design and five were single‐arm case series.

### 3.2 Study Characteristics

Study characteristics are summarized in Table 1. The studies originated from East Asia, Europe, the Middle East, and North America. Sample sizes ranged from 30 to 823 patients. Mean patient age ranged between 28 and 56 years, with a predominance of female participants across all cohorts. Minimum follow‐up duration ranged from 3 months to over 31 months, depending on the study.

### 3.3 Patient Characteristics

Primary indications included elongated philtrum, reduced vermilion show, diminished dental show, downturned oral commissures, and combined aesthetic concerns of the midface addressed concurrently with rhinoplasty. None of the included studies involved congenital deformities, trauma, or reconstructive indications.

### 3.4 Quantitative Outcomes

Quantitative morphometric and aesthetic outcomes are summarized in Table 2.

**TABLE 2:** Quantitative morphometric and aesthetic outcomes.

Study	Technique	Philtral length	Vermilion height	Dental/incisal show	Other measures	PROMs
Talei 2019 [8]	Modified deep‐plane	NR	NR	NR	NR	Qualitative improvement
Jeong 2020 [12]	Corner mouth lift	NR	NR	NR	NR	Qualitative
Jung 2019 [13]	Subnasal lift + tip plasty	L1 ratio 0.43→0.32	↑ qualitative	NR	NLA 91.31°→105.62°; ULA 48.97°→38.21°	High satisfaction
Buhsem 2023 [10] (G1)	Classical bullhorn	Excision 4–10 mm	NR	NR	NR	GAIS reported
Buhsem 2023 [10] (G2)	Dermal suspension	Excision 4–10 mm	NR	NR	NR	GAIS reported
Mahmood 2025 (Tri‐Lift) [11]	Deep‐plane suspension	14.50→10.75 mm	6.00→9.00 mm	2.00→5.00 mm	—	GAIS reported
Mahmood 2025 (Control) [11]	Traditional bullhorn	14.02→12.03 mm	5.05→7.01 mm	1.51→3.50 mm	—	GAIS reported
Pascali 2021 [14]	Indirect lift	23.5% shortening	NR	NR	NLA width + 10.9%	GAIS 4.4/5
Seo 2023 [15]	Central lip lift	NR	ULR 0.76→0.84; VUL 0.30→0.39	NR	LTW 0.34→0.39; CLA 101.59°→95.04°	NR

#### 3.4.1 Subnasal Bullhorn and Modified Bullhorn Techniques

- Mahmood (Tri‐Lift): philtral length decreased 14.50→10.75 mm; vermilion height increased 6.00→9.00 mm; dental show increased 2.00→5.00 mm.
- Mahmood (traditional bullhorn): philtral length decreased 14.02→12.03 mm; vermilion height increased 5.05→7.01 mm; dental show increased 1.51→3.50 mm.
- Buhsem (classical vs. dermal suspension): excision height 4–10 mm (mean not reported); no standardized anthropometric measurements.
- Talei: no quantitative measurements reported.

#### 3.4.2 Central Lip Lift

- Seo: ULR ratio increased 0.76→0.84; VUL ratio 0.30→0.39; LTW ratio 0.34→0.39; CLA changed 101.59°→95.04°.

#### 3.4.3 Subnasal Lip Lift With Nasal Tip Modification

- Jung: L1 ratio decreased 0.43 ± 0.05→0.32 ± 0.05; nasolabial angle increased 91.31°→105.62°; upper lip angle decreased 48.97°→38.21°.

#### 3.4.4 Combined Rhinoplasty and Indirect Lip Lift

- Pascali: lip length decreased by 23.5% at 1 year; nasolabial angle width increased by 10.9%.

#### 3.4.5 Corner Mouth Lift

- Jeong: no standardized pre‐/post‐operative morphometric values reported.

### 3.5 Patient‐Reported Outcomes

Five studies reported patient‐reported aesthetic outcomes. Two comparative studies provided numerical GAIS values, whereas others presented descriptive statements without standardized scales.

### 3.6 Complications

Complications are summarized in Table 3. No major complications were reported in any of the included studies. Minor complications included postoperative edema, bruising, hypertrophic scarring, transient dysesthesia, and localized epidermolysis. Revision procedures were reported in four studies, ranging from 0.6% to 6.7%. Two studies did not provide numeric revision rates.

**TABLE 3:** Complications and revision rates.

Study	*N*	Major complications	Minor complications	Revision rate
Talei 2019 [8]	823	None	Hematoma (*n* = 2); prolonged edema (*n* = 2); telangiectasia (*n* = 2); epidermolysis (*n* = 2); allergic rash (*n* = 10)	0.6%
Jeong 2020 [12]	498	None	Hypertrophic scar 4.6%; asymmetry; swelling	3.4%
Jung 2019 [13]	30	None	Swelling; dysesthesia; incisional redness 10%	6.7%
Buhsem 2023 [10]	28	None	NR	3.6%
Mahmood 2025 [11]	243	None	Swelling (quantitative NR)	NR
Pascali 2021 [14]	45	None	NR	NR
Seo 2023 [15]	87	None	NR	NR

### 3.7 Methodological Quality and Risk of Bias

Methodological quality assessment using the MINORS instrument demonstrated that all included studies were of moderate quality at best, with none meeting criteria for high methodological rigor (Table 4). Comparative studies achieved scores ranging from 18 to 20 out of 24, while single‐arm case series scored between 10 and 12 out of 16.

**TABLE 4:** Methodological quality (MINORS).

Study	MINORS score	Quality classification
Mahmood 2025 [11]	20/24	Moderate–good
Buhsem 2023 [10]	18/24	Moderate
Talei 2019 [8]	10/16	Moderate
Jeong 2020 [12]	10/16	Moderate
Jung 2019 [13]	12/16	Moderate
Pascali 2021 [14]	11/16	Moderate
Seo 2023 [15]	10/16	Moderate

Importantly, no randomized controlled trials were identified, and none of the included studies reported blinded outcome assessment or prospective sample size or power calculations. Follow‐up duration varied substantially across studies, and handling of loss to follow‐up was inconsistently reported.

These methodological limitations introduce a measurable risk of bias and directly affect the strength of the available evidence. In particular, the absence of blinding increases susceptibility to observer bias, while the lack of prospective study design and power calculation limits confidence in the magnitude and precision of reported effects.

Consequently, although consistent directional improvements were observed across studies, the methodological quality of the evidence constrains the reliability of comparative interpretation between different lip lift techniques.

## 4 Discussion

This systematic review synthesizes the contemporary evidence on aesthetic upper‐lip lifting techniques and demonstrates a consistent pattern of improvement in philtral height, vermilion display, and perioral proportions across all procedures examined. Despite notable heterogeneity in study design and outcome reporting, the direction of effect was uniform: no study reported paradoxical philtral lengthening, reduced vermilion show, or diminished dental exposure after surgery [9, 10, 11, 12, 13, 14, 15]. These consistent qualitative outcomes suggest that properly executed lip lift procedures reliably restore youthful vertical proportions of the upper lip.

Among the techniques with quantitative documentation, the deep‐plane Tri‐Lift and the traditional subnasal bullhorn demonstrated the most robust millimetric improvements [9]. When expressed as ratios or angular measurements, central lip lift [12] and subnasal lift combined with nasal tip modification [11] also showed clear proportional enhancement of upper‐lip aesthetics. The indirect rhinoplasty‐based lip lift described by Pascali et al. [15] produced moderate but measurable reductions in lip length (23.5% at 1 year), confirming that even indirect perinasal manipulation can influence upper‐lip morphology.

Taken together, the available data do not identify a single universally superior technique; rather, they allow for a deformity‐specific, algorithmic approach that tailors the choice of method to the dominant anatomical imbalance.

### 4.1 Comparison of Techniques and Their Strengths

#### 4.1.1 Deep‐Plane Tri‐Lift

The deep‐plane Tri‐Lift demonstrated the largest combined improvements in philtral height, vermilion show, and dental exposure in the only comparative study available [9]. The incorporation of sub‐SMAS release and muscular suspension likely contributes to enhanced excursion, eversion, and stability. However, these findings are limited to one center and require validation in independent cohorts.

#### 4.1.2 Traditional Subnasal Bullhorn and Modifications

The bullhorn lift remains the most widely published procedure, with predictable vertical shortening and typically favorable scar concealment [9, 10, 14]. Dermal‐suspension variants [10] and extended, alar‐base modifications appear intended to optimize tension distribution and longevity, but quantitative proof of superiority remains insufficient.

#### 4.1.3 Central Lip Lift

Seo et al. [15] reported improvements predominantly in central vermilion exposure and midline ratios (ULR, VUL, LTW). This technique is best suited to patients with predominant midline deficiency rather than generalized philtral elongation.

#### 4.1.4 Subnasal Lift With Nasal Tip Modification

The combined approach described by Jung et al. [13] effectively modified the L1 ratio and nasal–labial parameters and may be appropriate when nasal tip ptosis coexists with upper‐lip elongation.

#### 4.1.5 Indirect Lip Lift During Rhinoplasty

The technique proposed by Pascali et al. [14] provides modest but functionally meaningful refinement without a direct subnasal incision. It may be a reasonable adjunct in patients undergoing primary or revision rhinoplasty with mild lip lengthening.

#### 4.1.6 Corner Mouth Lift

The corner lift addresses age‐related commissural descent and should be viewed as complementary to, not a substitute for, philtral diminishment procedures [13].

### 4.2 Safety, Complications, and Revision Rates

Across all studies, no major complications were reported [9, 10, 11, 12, 13, 14, 15]. Minor complications—edema, bruising, transient dysesthesia, hypertrophic scarring, epidermolysis, telangiectasia, and mild asymmetry—were generally self‐limiting and expected within the spectrum of perioral soft‐tissue surgery.

Hypertrophic scarring was quantified only in the corner lift series (4.6%) [13], whereas other studies either did not specify scar outcomes or described them qualitatively. Revision rates ranged from 0.6% (deep‐plane series) to 6.7% (combined subnasal lift + tip plasty) [9, 10, 11, 14, 15]. Most revisions addressed aesthetic refinements rather than structural failures.

Given the lack of standardized scar assessment tools and the variability in follow‐up (3–31 months), long‐term stability remains insufficiently characterized.

### 4.3 Proposed Expert‐Informed Decision‐Making Framework

The following framework represents an expert‐informed interpretive model rather than a comparative evidence‐based guideline. Although it is informed by patterns observed across the included studies, it is not derived from high‐level comparative evidence. Importantly, no randomized or controlled head‐to‐head studies directly comparing different lip lift techniques—such as central lip lift versus subnasal bullhorn lift for specific indications like central vermilion insufficiency—were identified in this review.

Accordingly, the following recommendations represent an expert‐informed synthesis of limited and heterogeneous evidence, primarily extrapolated from single‐arm observational studies and established surgical principles, rather than evidence‐based clinical guidelines.

Based on the available data, it is not methodologically sound to claim one technique as universally superior. However, the consistency and magnitude of the reported outcomes allow for an evidence‐informed, deformity‐driven interpretive framework:

Generalized philtral elongation with reduced incisal show: Deep‐plane Tri‐Lift or traditional subnasal bullhorn techniques provide the most predictable vertical shortening and improvement in dental exposure.

Dominant central vermilion insufficiency with preserved lateral architecture: Central lip lift may offer targeted enhancement of midline vermilion display without excessive lateral elevation.

Concurrent nasal tip ptosis or rhinoplasty‐associated imbalance: Subnasal lift combined with nasal tip modification or indirect rhinoplasty‐based lip lift may be appropriate in selected cases.

Downturned oral commissures or perioral aging pattern: Corner mouth lift should be considered as an adjunctive procedure rather than a substitute for philtral shortening techniques.

Thus, the concept of an “optimal” technique should be understood as diagnosis‐specific rather than universal, with final technique selection guided by individual anatomical characteristics, surgeon experience, and intraoperative judgment.

### 4.4 Preoperative Evaluation and Planning

Although the included studies did not provide standardized preoperative protocols, their use of cephalometric ratios and anthropometric measurements underscores the need for a structured preoperative workflow [9, 10, 11, 12, 13, 14, 15]. A comprehensive evaluation should include:

- standardized frontal, oblique, and profile photography at rest and smiling;
- measurements of philtral height, vermilion height, dental/incisal show;
- angular assessment (nasolabial angle, upper‐lip angle, columella–labial angle);
- ratio analysis when appropriate (ULR, VUL, LTW, L1) [12, 13];
- evaluation of skin type (risk of hypertrophic scarring), prior fillers, implants, or perioral surgery;
- assessment of dental and skeletal support, particularly maxillary retrusion.

Clear documentation of planned numeric corrections (mm or ratios) facilitates surgical precision and postoperative interpretation.

### 4.5 Intraoperative Considerations: Incision Design, Closure, and Suture Strategy

The included studies did not systematically compare closure methods or suture materials [9, 10, 11, 12, 13, 14, 15], representing a significant evidence gap. Nevertheless, based on established principles of facial plastic surgery and the complication patterns reported, several practical recommendations can be derived:

- Layered closure is essential. Buried deep dermal sutures (5–0 or 6–0 absorbable) reduce superficial tension.
- Cutaneous closure should be performed with fine materials, such as 6–0 or 7–0 nylon or polypropylene, or fast‐absorbing gut in select cases.
- Avoid excessive epidermal tension, which correlates with widened scars and contour irregularities.
- Meticulous alignment at the alar base is critical to prevent notching, asymmetry, or distortion of the nostril sill.

These recommendations should be considered expert‐level guidance rather than evidence‐based directives, pending comparative studies.

### 4.6 Postoperative Care and Scar Management

Postoperative protocols were poorly reported, yet the pattern of complications provides insight into optimal care strategies:

- head elevation and cold compresses for 48–72 h to reduce edema;
- avoidance of excessive upper‐lip movement (laughing, wide mouth opening) during early wound healing;
- removal of cutaneous sutures between day 5–7 to minimize track marks;
- early initiation (after epithelialization) of silicone gel, silicone sheeting, or micropore taping to prevent hypertrophic scarring;
- strict photoprotection for at least 3 months;
- monitoring for telangiectasia or persistent dysesthesia.

Given the aesthetic sensitivity of the region, future studies should evaluate whether specific regimens demonstrably reduce adverse scar outcomes.

### 4.7 Methodological Limitations

Although the review protocol was prospectively registered in PROSPERO and all analyzes were conducted in accordance with predefined objectives and methodology, several important limitations of the current evidence base must be acknowledged.

All included studies were observational in design, with no randomized or controlled comparative trials available [9, 10, 11, 12, 13, 14, 15]. Outcome reporting was markedly heterogeneous, with results expressed using absolute millimeter measurements, ratios, or percentage changes, limiting cross‐study comparability.

Additional limitations include the absence of three‐dimensional or AI‐based morphometric analysis, variable and often short follow‐up periods, lack of blinded aesthetic assessment, absence of stratification by gender, ethnicity, or skeletal pattern, and limited evaluation of scar‐related risk factors.

Collectively, these methodological weaknesses restrict the strength of the available evidence and preclude the formulation of definitive, technique‐specific, evidence‐based guidelines. Consequently, the conclusions of this review should be interpreted as an expert‐informed synthesis of limited and heterogeneous data rather than high‐level comparative evidence.

### 4.8 Clinical Implications

Despite methodological constraints, the existing evidence supports the safety and effectiveness of upper‐lip lift procedures in carefully selected patients. Surgeons should:

- adopt a deformity‐driven approach to technique selection;
- prioritize thorough preoperative morphometric evaluation;
- employ layered closure techniques to optimize scar outcomes;
- counsel patients regarding typical improvements and possible revision risk (0.6%–6.7%);
- emphasize postoperative scar management.

### 4.9 Future Research Directions

Future research in upper lip lift surgery should prioritize methodologically robust, comparative study designs to address the substantial gaps identified in the current literature. In particular, prospective randomized controlled trials comparing contemporary deep‐plane techniques with traditional subnasal bullhorn approaches are needed to determine whether claimed advantages in longevity, scar quality, and dynamic lip motion are supported by high‐quality evidence.

Such studies should incorporate standardized and reproducible outcome measures, including objective three‐dimensional photogrammetry to quantify changes in philtrum length, vermilion height, and nasolabial angle over time. Equally important is the consistent use of validated patient‐reported outcome measures, such as the FACE‐Q Lip module, to allow meaningful comparison of patient satisfaction and perceived aesthetic benefit across techniques.

Future investigations should also include sufficiently long follow‐up periods to evaluate the durability of surgical outcomes and the incidence of late complications or revisions, which were inconsistently reported in the available studies. Additionally, stratification by patient characteristics—such as age, skin type, baseline philtrum length, and prior perioral filler exposure—may help refine patient selection criteria and improve individualized surgical planning.

Finally, multicenter collaborative studies with standardized reporting frameworks would enhance external validity and facilitate the development of evidence‐informed consensus recommendations for technique selection in upper lip lift surgery.

## 5 Conclusion

Current evidence confirms that lip lift surgery is a safe and effective option for restoring youthful upper‐lip proportions, with consistent directional improvements across all techniques. While no single method can be universally recommended, a deformity‐specific algorithm—grounded in careful preoperative evaluation and evidence‐informed perioperative care—provides the most rational framework for technique selection. Robust comparative studies with standardized measurements are essential before definitive, high‐level guidelines can be established.

## Author Contributions

**Oskar Komisarek**, **Łukasz Banasiak:** conceptualization. **Oskar Komisarek**, **Vanessa Olichwer**, **Paweł Burduk:** methodology. **Oskar Komisarek**, **Łukasz Banasiak**, **Vanessa Olichwer**, **Paweł Burduk:** validation. **Oskar Komisarek**, **Łukasz Banasiak**, **Vanessa Olichwer:** formal analysis. **Oskar Komisarek**, **Łukasz Banasiak**, **Vanessa Olichwer:** investigation. **Oskar Komisarek**, **Łukasz Banasiak**, **Vanessa Olichwer**, **Paweł Burduk:** resources. **Oskar Komisarek**, **Łukasz Banasiak**, **Vanessa Olichwer**, **Paweł Burduk:** data curation. **Oskar Komisarek**, **Łukasz Banasiak**, **Vanessa Olichwer**, **Paweł Burduk:** writing – original draft. **Oskar Komisarek**, **Łukasz Banasiak**, **Vanessa Olichwer**, **Paweł Burduk:** writing – review and editing. **Oskar Komisarek:** visualization. **Paweł Burduk:** supervision. **Oskar Komisarek:** project administration.

## Funding

The authors have nothing to report.

## Ethics Statement

The authors have nothing to report.

## Conflicts of Interest

The authors declare no conflicts of interest.

## Supporting information


**Table S1:** Complete and reproducible search strategies.

## Data Availability

Data sharing not applicable to this article as no datasets were generated or analyzed during the current study.
