# Supplementary material for: The Bullhorn and Beyond: Evidence‐Based Review and Clinical Recommendations for Lip Lift Techniques
Source: J Cosmet Dermatol. 2026 Feb 23;25(3):e70703. doi: 10.1111/jocd.70703 (PMC12929697; doi:10.1111/jocd.70703)
Supplement: Supplementary file 1 — Table S1: Complete and reproducible search strategies. [file JOCD-25-e70703-s001.docx]

Supplementary Appendix

Table S1 Complete and Reproducible Search Strategies

| Database | Search strategy | Limits / filters | Date of last search |
| --- | --- | --- | --- |
| PubMed/MEDLINE | ("lip lift" OR "subnasal lift" OR "bullhorn lip lift" OR "deep-plane lip lift" OR "Italian lip lift" OR "corner lip lift") AND ("aesthetic" OR "facial rejuvenation" OR "perioral") | Language: English; Publication dates: January 1, 2017 – October 31, 2025 | October 31, 2025 |
| Google Scholar | ("lip lift" OR "upper lip lift" OR "subnasal lip lift" OR "bullhorn lip lift" OR "deep-plane lip lift" OR "Italian lip lift" OR "corner lip lift") AND aesthetic | Language: English; Publication years: 2017–2025 | October 31, 2025 |
| Web of Science | TS=("lip lift" OR "upper lip lift" OR "subnasal lip lift" OR "bullhorn lip lift" OR "deep-plane lip lift" OR "Italian lip lift" OR "corner lip lift") AND TS=(aesthetic* OR cosmetic* OR "facial rejuvenation" OR perioral) | Language: English; Publication years: 2017–2025 | October 31, 2025 |
| Scopus | TITLE-ABS-KEY ("lip lift" OR "upper lip lift" OR "subnasal lip lift" OR "bullhorn lip lift" OR "deep-plane lip lift" OR "Italian lip lift" OR "corner lip lift") AND TITLE-ABS-KEY (aesthetic* OR cosmetic* OR "facial rejuvenation" OR perioral) | Language: English; Publication years: 2017–2025 | October 31, 2025 |
| Cochrane Library | ("lip lift" OR "upper lip lift" OR "subnasal lip lift" OR "bullhorn lip lift" OR "deep-plane lip lift" OR "Italian lip lift" OR "corner lip lift") in Title Abstract Keyword | Publication years: 2017–2025 | October 31, 2025 |
